# Dynamical systems analysis of spike-adding mechanisms in transient bursts

**DOI:** 10.1186/2190-8567-2-7

**Published:** 2012-04-24

**Authors:** Jakub Nowacki, Hinke M Osinga, Krasimira Tsaneva-Atanasova

**Affiliations:** 1 Bristol Centre for Applied Nonlinear Mathematics, Department of Engineering Mathematics, University of Bristol, Queen’s Building, University Walk, Bristol, BS8 1TR, United Kingdom; 2 Department of Mathematics, The University of Auckland, Private Bag 92019, Auckland, 1142, New Zealand

**Keywords:** burst, spike adding, transient behaviour, dynamical systems, geometric singular perturbation theory

## Abstract

Transient bursting behaviour of excitable cells, such as neurons, is a common feature observed experimentally, but theoretically, it is not well understood. We analyse a five-dimensional simplified model of after-depolarisation that exhibits transient bursting behaviour when perturbed with a short current injection. Using one-parameter continuation of the perturbed orbit segment formulated as a well-posed boundary value problem, we show that the spike-adding mechanism is a canard-like transition that has a different character from known mechanisms for periodic burst solutions. The biophysical basis of the model gives a natural time-scale separation, which allows us to explain the spike-adding mechanism using geometric singular perturbation theory, but it does not involve actual bifurcations as for periodic bursts. We show that unstable sheets of the critical manifold, formed by saddle equilibria of the system that only exist in a singular limit, are responsible for the spike-adding transition; the transition is organised by the slow flow on the critical manifold near folds of this manifold. Our analysis shows that the orbit segment during the spike-adding transition includes a fast transition between two unstable sheets of the slow manifold that are of saddle type. We also discuss a different parameter regime where the presence of additional saddle equilibria of the full system alters the spike-adding mechanism.

## 1 Introduction

 How a single spike or a burst of spikes is generated and regulated for neuron cells is one of the most fundamental questions in neuroscience [1]. Spike generation is closely related to neuronal excitability, which is the ability of the cell’s membrane potential to undergo a large excursion, called an action potential or a spike, when subjected to a sufficiently strong stimulus [1-3]. An excitable cell either responds in full to such a stimulus or not at all, which allows for a reliable transmission of information. Therefore, the different mechanisms for excitability and bursting have been widely studied; we refer to Izhikevich [4] for a comprehensive overview of mechanisms for neurons. Excitable behaviour has also been reported to occur in many other types of cells [1-3] as well as in physical systems, such as lasers [5,6], electronic circuits [7-10] and chemical reactions [11]. Neuronal excitability can be very sensitive to even relatively small changes in, for example, the biophysical properties of a neuron [12-14] or its morphology [15]. This parameter sensitivity indicates that dynamical systems theory is particularly suited for explaining the rich dynamics found in excitable systems.

 The classification of different bursting mechanisms was pioneered by Rinzel [16], who used a system decomposition into slow and fast subsystems. He showed that the burst can be divided into active (spiking) and silent phases, which follow different types of attractors of the fast subsystem. Hence, a classification of the bursting oscillators is provided by the structure of the bifurcation diagram of the fast subsystem. Rinzel’s classification was extended in Izhikevich’s studies [1,4]. As an alternative approach, Golubitsky et al. [17] used singularity theory to classify the bifurcation diagrams of the fast subsystem and, hence, the different bursting mechanisms; see also [18]. These studies are primarily for systems with one slow variable; see Smolen et al. [19] for an extension to two slow variables and Ermentrout and Terman [3] for a summary of these ideas along with new results.

 A classification of bursting mechanisms, however, does not answer questions about the number of spikes in a particular burst of the same type nor does it explain possible transitions between spiking and bursting. While the latter has received considerable attention over the years, the former has hardly been addressed. Terman [20] analysed transitions between bursting and tonic (continuous) spiking in a pancreatic *β*-cell model. He recognised the importance of connecting classical slow-fast analysis with full system bifurcation analysis and identified bifurcations of the periodic bursting solutions that organise the transitions between different parameter regimes. He further studied chaotic spiking that can arise in between such transitions [21]. Recently, Benes et al. [22] and Kramer et al. [23] reported that a new type of torus canard can play a role in the transition from spiking to bursting in a model of Purkinje cells. Other recent studies particularly focus on spike-adding in a periodic bursting oscillator; for example, see Govaerts and Dhooge [24], Guckenheimer and Kuehn [25], Tsaneva-Atanasova et al. [26] and Linaro et al. [27]. These studies show that the spike-adding mechanism is formed by a pair of saddle-node bifurcations of periodic orbits of the full system; bursts with different numbers of spikes are, in fact, different periodic attractors of the full system that may coexist only if the number of spikes differs by one [20,26]. The recent study by Teka et al. [28] explains how one may predict the precise number of spikes in a burst; here, the use of two slow variables is essential. Methods to regulate the number of spikes are reported by Ghigliazza and Holmes [29], where a minimal Hodgkin-Huxley model of bursting is proposed to analyse spike-adding and transitions between bursting and tonic spiking in a more general context.

 As discussed by Izhikevich [4], the mechanisms for generating spikes do not depend on whether the neuron exhibits spiking only as a transient phenomenon when subjected to a strong enough stimulus or whether it is spiking or bursting continuously. This is indeed the case if transient bursting is organised by the applied stimulus. Applying a stimulus has the effect of changing the right-hand side of the underlying system of ODEs. The bifurcation diagram of the corresponding fast subsystem typically no longer has a stable equilibrium that corresponds to the resting potential, and a spike or burst arises from new attractors that exist only when the stimulus is on. As soon as the stimulus is switched off, the system relaxes back to the resting potential. Therefore, the mechanism is due to a change in the structure of the bifurcation diagram, which depends on the strength of the applied stimulus. Studies of this nature, where the type of burst is studied in dependence on the strength or duration of the stimulus, have been done, for example, by Tran et al. [30], Kim et al. [31] and Stern et al. [32].

 In this paper, we investigate spike-adding in a transient burst in the model of hippocampal pyramidal neurons from Nowacki et al. [13]. In contrast to the above studies, the spike-adding occurs *after* the applied stimulus has been switched off. Hence, the bursting behaviour is governed by the underlying bifurcation structure of the original system, for which a stable equilibrium exists. The strength of the applied stimulus must be such that a first action potential is generated, but the stimulus is not responsible for generating any additional spikes. Our investigation can be compared to excitability in laser systems [5,6] where the response after an applied stimulus is explained by the existence of a nearby homoclinic bifurcation with respect to a parameter. This mechanism is due to the presence of a saddle equilibrium that coexists with the stable equilibrium (i.e., the resting potential). The interesting aspect about our model is that there exists only a stable equilibrium, and we have been unable to identify any nearby saddle-type invariant object in the parameter region of interest that could organise homoclinic bifurcations. We explain the transient behaviour following the ideas of Geometric Singular Perturbation Theory (GSPT) [33-36].

 Another important difference with existing studies is that we do not rely on simulations to study the effects of an applied stimulus in numerical experiments. Instead, we use a continuation-based approach and show the process of spike-adding transitions during a transient burst in unprecedented detail, which is not possible using brute-force simulation. Our numerical method is based on the continuation of orbit segments as solutions of a two-point boundary value problem; this approach has already been applied to the bifurcation analysis of periodic orbits, including homoclinic or heteroclinic bifurcations [37], and more recently for the computation of invariant manifolds [38] and so-called slow manifolds in systems with multiple time scales [39,40]. We divide the system into two separate orbit segments, with and without current injection, which are coupled only by the boundary conditions. This allows us to continue the orbit segments in a chosen parameter and analyse the precise nature of their continuous deformation even over exponentially small parameter variations. We complement this continuation with the computation of the two-dimensional *critical manifold* of the fast subsystem, which comprises all the equilibria of the fast subsystem parametrised by two slow variables. The critical manifold is folded and consists of a number of stable and unstable sheets. In our setting, the unstable sheets are of saddle type, and we will refer to them as *saddle-unstable* sheets. The fast subsystem also has families of periodic orbits that emanate from Hopf bifurcations on the critical manifold, which give rise to the spiking behaviour during a burst. Our analysis shows that, during the spike-adding transition, orbit segments trace saddle-unstable slow manifolds that lie very close to corresponding saddle-unstable sheets of the critical manifold; the distance between these two manifolds is of the same order as the ratio between the contraction/expansion rates towards and on the manifold, which is organised by the difference between the slow and fast time scales [33,41]. This canard-like behaviour is very similar to behaviour during a spike-adding transition of a periodic burst [25], but it does not involve bifurcations, and coexistence of bursts with different numbers of spikes is not possible here. Thus, our analysis indicates that it is the presence of canard-like behaviour that organises the spike adding.

 As for periodic bursting, the spike-adding transition in our model occurs over an exponentially small parameter interval [42-44]. Within this exponentially small parameter interval, we find an even smaller parameter interval during which the canard-like orbit segment includes a fast transition from a saddle-unstable to *another saddle-unstable* slow manifold. This phenomenon is similar to the so-called fold-initiated canards that have been observed for periodic orbits [45]. To understand this behaviour, we study the associated slow flow on the critical manifold and identify the effect of folds and folded singularities on the behaviour of the orbit segment. Our findings complement the study for planar systems by Guckenheimer et al. [45] and confirm that such fold-initiated canard-like behaviour occurs robustly during a spike-adding transition due to continuity of the vector field with respect to the parameter.

 Our study concerns the analysis of a spike-adding mechanism where the full system has a unique stable equilibrium that does not undergo any bifurcations. We find that, for different values of model parameters, the system can have additional unstable equilibria that alter the nature of the spike-adding mechanism. More precisely, the appearance of two saddle equilibria on the critical manifold suppresses the fold-initiated transition between saddle-unstable sheets and changes the behaviour of the orbit segment. The presence of the saddle equilibria give rise to a mechanism that is reminiscent of the excitability studies by Wieczorek et al. [5] and Krauskopf et al. [6]; a new spike is added via a heteroclinic connection of the perturbed state to a saddle equilibrium of the full system. However, this saddle equilibrium lies outside a neighbourhood of the resting potential, and we observe a canard-like transition before and after the heteroclinic connection that is similar to the canard-like transition that occurs when no additional equilibria are present. We compute the critical manifold for this situation and study the associated slow flow to explain this phenomenon; we find that the saddle equilibrium point lies in the middle of a saddle-unstable sheet of the critical manifold, and it is an attractor with respect to the slow flow on the critical manifold.

This paper is organised as follows: In the next section we present our model of the study. In Section 3, we numerically identify the mechanism of spike-adding via continuation of the orbit segment. Next, in Section 4, we calculate the critical manifold of the fast subsystem along with the slow flow to explain the transition to bursting. Here, we also investigate the transition between two unstable slow manifolds of the saddle type and the changes in the spike-adding mechanism when additional equilibria of the full system are present. We end with a discussion in Section 5.

## 2 The model

 We apply our analysis of transient bursts to pyramidal neuron cells from the CA1 and CA3 regions of the hippocampus. A detailed model of such neurons in Hodgkin-Huxley formalism was presented by Nowacki et al. [13], but for the purpose of this paper, we study a reduced version of this model. The simplified model consists of four ionic currents, namely, fast and slow inward currents, denoted as $I_{\mathrm{FI}}$ and $I_{\mathrm{SI}}$, respectively, and fast and slow outward currents, denoted as $I_{\mathrm{FO}}$ and $I_{\mathrm{SO}}$, respectively. Inward currents are responsible for the depolarisation or increase of the membrane potential, whereas outward currents hyperpolarise or decrease the membrane potential and return the cell back to its resting state (a stable equilibrium) [2,46]. The fast inward current $I_{\mathrm{FI}}$ represents the fastest class of spike-generating ${\mathrm{Na}}^{+}$- and ${\mathrm{Ca}}^{2+}$-currents. The rates of change of these currents are usually similar to that of the membrane potential. Therefore, we assume that the gating of $I_{\mathrm{FI}}$ is instantaneous [13,46-48]. The slow inward current $I_{\mathrm{SI}}$ mainly corresponds to the transient T-type ${\mathrm{Ca}}^{2+}$-current [13,49-51] and represents the low-voltage activated currents responsible for shaping the subthreshold behaviour of the model. The fast outward current $I_{\mathrm{FO}}$ represents high-voltage activated fast $K^{+}$-currents that we base on the delayed rectifier $K^{+}$-current [13,46,47]. Finally, $I_{\mathrm{SO}}$ represents muscarinic-sensitive $K^{+}$-current [50,52,53], which has an activation rate of the same order as that of $I_{\mathrm{SI}}$.

We only consider the following variables as dynamic: the membrane potential *V*, the gating variables $m_{\mathrm{SI}}$, $m_{\mathrm{FO}}$ and $m_{\mathrm{SO}}$ that govern activation of the respective currents and the gating variable for inactivation of $I_{\mathrm{SI}}$, which we denote by $h_{\mathrm{SI}}$. Hence, our reduced model is five dimensional and has the form

(1) $$\frac{du}{dt}=\frac{d}{dt}\left(\begin{array}{c} V\\ m_{\mathrm{SI}}\\ m_{\mathrm{FO}}\\ m_{\mathrm{SO}}\\ h_{\mathrm{SI}} \end{array}\right)=f(u,\lambda ,I_{\mathrm{app}}):=\left(\begin{array}{c} f_{1}(u,\lambda ,I_{\mathrm{app}})\\ f_{2}(u,\lambda )\\ f_{3}(u,\lambda )\\ f_{4}(u,\lambda )\\ f_{5}(u,\lambda ) \end{array}\right).$$

Here, $u\in R^{5}$ is the non-dimensionalised state vector, and $I_{\mathrm{app}}$ is an applied current that stimulates (perturbs) the cell model when it is non-zero. We specifically indicate further parameter dependencies with the parameter vector $\lambda \in R^{k}$ for some integer k>0. In this paper, we primarily focus on how the system depends on the maximal conductances of $I_{\mathrm{SI}}$ and $I_{\mathrm{FO}}$; these parameters are likely to vary between neurons due to different sizes and numbers of channels in different cells (even among the same types of neurons). The right-hand side of (1) has the specific form that is well known from Hodgkin-Huxley formalism: the dynamics of the membrane potential is organised by the equations for the ionic currents, modelled as

(2) $$\begin{array}{rcl} C_{m}\frac{dV}{dt} & = & -(I_{\mathrm{FI}}+I_{\mathrm{SI}}+I_{\mathrm{FO}}+I_{\mathrm{SO}})+I_{\mathrm{app}}\\ & = & -(g_{\mathrm{FI}}m_{{\mathrm{FI}}_{\infty }}(V)(V-E_{I})+g_{\mathrm{SI}}m_{\mathrm{SI}}^{2}h_{\mathrm{SI}}(V-E_{I})\\ & & +g_{\mathrm{FO}}m_{\mathrm{FO}}(V-E_{O})+g_{\mathrm{SO}}m_{\mathrm{SO}}(V-E_{O}))+I_{\mathrm{app}}, \end{array}$$

where $C_{m}$ is the membrane capacitance. Here, $g_{x}$ with x∈{FI,SI,FO,SO} are maximal conductances of the currents, and $E_{I}$ and $E_{O}$ are Nernst potentials of the inward and outward currents, respectively. Note that $I_{\mathrm{FI}}$ only depends on *V*, that is, $m_{\mathrm{FI}}=m_{{\mathrm{FI}}_{\infty }}(V)$ as defined in (4) below. The dynamics of the gating variables is modelled by

(3) $$\frac{dx}{dt}=\frac{x_{\infty }(V)-x}{\tau_{x}},\;\text{where }x\in \{m_{\mathrm{SI}},m_{\mathrm{FO}},m_{\mathrm{SO}},h_{\mathrm{SI}}\};$$

the corresponding activation and inactivation steady-state functions $x_{\infty }(V)$ of the respective currents, as well as $m_{{\mathrm{FI}}_{\infty }}(V)$, are given in Boltzmann form as:

(4) $$x_{\infty }(V)=\frac{1}{1+\exp(-\frac{V-V_{x}}{k_{x}})}.$$

Unless specified otherwise, the default values that we use for the parameters of this simplified model are summarised in Table 1. 

**Table 1:** Parameter values for the simplified model (1) as defined in (2)-(4)

**Fig. 1:**
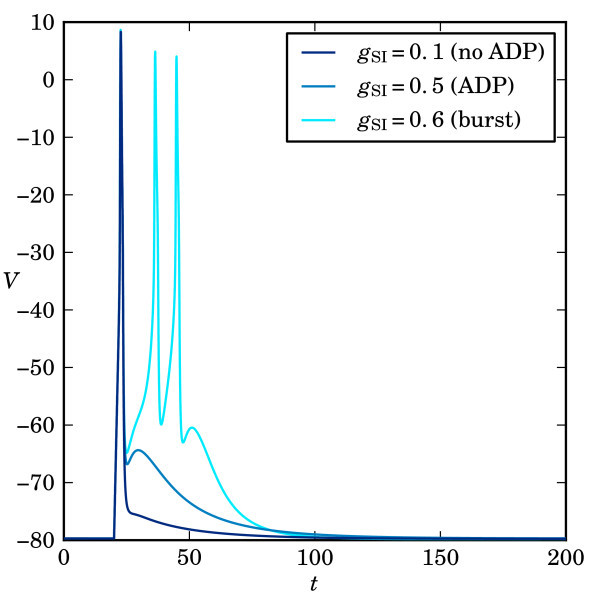
Responses of system (1) to a current injection of $I_{\mathrm{app}}=20\;\mu {\text{A/cm}}^{2}$. The current injection is applied from t=50 to t=53. Overlaid are the responses corresponding to different values of the maximal conductance $g_{\mathrm{SI}}$ (in mS/cm^2^) of the slow inward current, namely, $g_{\mathrm{SI}}=0.1$, $g_{\mathrm{SI}}=0.5$ and $g_{\mathrm{SI}}=0.6$, which are examples of responses with no ADP, with ADP and a three-spike burst with ADP, respectively.

 Figure 1 illustrates three classes of the responses of the simplified model obtained by changing the maximal conductance $g_{\mathrm{SI}}$. These correspond to cell responses that are typically observed experimentally. During the simulations, the model is perturbed from its stable equilibrium by a short current injection whose duration guarantees that the rapidly rising membrane potential will reach and cross its local maximum, creating a fully developed spike; see [12,13,47,48] for more details. Two of the three typical responses shown in Figure 1 exhibit a positive deflection of the membrane potential characterised by a ‘hump’ in the time trace of the membrane potential at the end of the burst; this is called after-depolarisation (ADP), which can exist provided that $\tau_{m_{\mathrm{FO}}}<\tau_{m_{\mathrm{SI}}}$[13]. Only the first response (lower curve) is a spike without ADP. Note that the last trace, which corresponds to $g_{\mathrm{SI}}=0.6$, the highest value of $g_{\mathrm{SI}}$ in the example, has sufficiently strong $I_{\mathrm{SI}}$ to enable the membrane potential to cross the excitability threshold during the ADP so that additional spikes are fired. 

 System (1) defined by Equations (2)-(4) evolves on multiple time scales because $C_{m}/g_{\mathrm{FO}}$ (as an approximation of the time scale for *V*) and $\tau_{x}$ with $x\in \{m_{\mathrm{SI}},m_{\mathrm{FO}},m_{\mathrm{SO}},h_{\mathrm{SI}}\}$ have different orders of magnitude. As indicated in Table 1$m_{\mathrm{SO}}$ and $h_{\mathrm{SI}}$ are slow variables that vary on a time scale that is (roughly) 10 times slower than $m_{\mathrm{SI}}$ and $m_{\mathrm{FO}}$ and 100 times slower than *V*. In particular, this means that our model is capable of firing an arbitrarily large number of spikes during the ADP. More precisely, an increase in $g_{\mathrm{SI}}$, as in Figure 1 and throughout this paper, has the net effect that the slow variable $h_{\mathrm{SI}}$ becomes even slower so that more spikes can be fired during the time it takes for $h_{\mathrm{SI}}$ to relax back to its equilibrium value. In this paper, we are not interested in the exact nature of this process, but we mention here that a large number of spikes will also be accompanied by a noticeable reduction in oscillation amplitudes. As $h_{\mathrm{SI}}$ slows down, the dynamics will resemble more and more the behaviour organised by slow passage through a Hopf bifurcation [26,54]. We focus on the process of spike adding and take advantage of the difference in time scales in Section 3, where it suffices to consider the time-scale separation between the three fast variables *V*$m_{\mathrm{SI}}$ and $m_{\mathrm{FO}}$, and the two slow variables $m_{\mathrm{SO}}$ and $h_{\mathrm{SI}}$.

 The gating variables express the fractions of channels in a given state and naturally range over the interval [0,1]. The natural range of the membrane potential *V* is bounded by the two Nernst potentials [2,46], i.e., $E_{O}\leq V\leq E_{I}$, where $E_{O}=-80.0\text{ mV}$ and $E_{I}=80.0\text{ mV}$. It is beneficial for the numerical analysis if all variables vary over a similar range. Therefore, the computations are done using the scaled membrane potential $V/k_{v}$, where $k_{v}=100\text{ mV}$. For our numerical investigations, we used the continuation package Auto[55,56] for solving the boundary value problems. All numerical simulations were done with XPP [57] using the front-end package XPPy [58] in Python [59], and visualisations were done in Python using Matplotlib [60] and Mayavi [61].

## 3 Identifying the spike-adding mechanism

Spike adding happens after a current injection, that is, in the regime where $I_{\mathrm{app}}=0$. Hence, any numerical investigation of the transient behaviour must take into account a discontinuous jump from $I_{\mathrm{app}}>0$ to $I_{\mathrm{app}}=0$ on the right-hand side of Equation (2). We view the orbit as a concatenation of two orbit segments that are the solution of two boundary value problems and define appropriate boundary conditions to account for the discontinuity in $I_{\mathrm{app}}$.

**Fig. 2:**
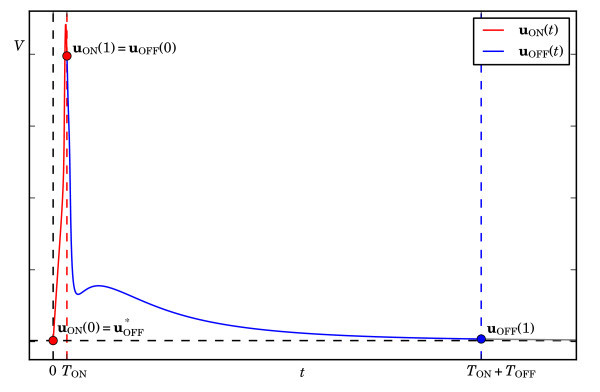
Formulation of system (1) as the boundary value problem (5)-(8). The first (*red*) segment is the solution $u_{\mathrm{ON}}(t)$ of (1) with $I_{\mathrm{app}}=20\;\mu {\text{A/cm}}^{2}$ and $u_{\mathrm{ON}}(0)$ at the resting potential $u_{\mathrm{OFF}}^{*}$, which is an equilibrium of (1) for $I_{\mathrm{app}}=0$ (indicated by the *horizontal black dashed line*). The total integration time is $T_{\mathrm{ON}}=3\text{ ms}$, such that one action potential occurs. The second (*blue*) segment is the solution $u_{\mathrm{OFF}}(t)$ of (1) with $I_{\mathrm{app}}=0$ and $u_{\mathrm{OFF}}(0)=u_{\mathrm{ON}}(1)$; the integration time $T_{\mathrm{OFF}}$ is fixed to a large enough value so that $u_{\mathrm{OFF}}(1)\approx u_{\mathrm{OFF}}^{*}$.

 More precisely, we consider two successive orbit segments denoted as $u_{\mathrm{ON}}$ and $u_{\mathrm{OFF}}$, during which current is injected ($I_{\mathrm{app}}>0$) and during which it is not ($I_{\mathrm{app}}=0$), respectively; the concatenation of the two orbit segments $u_{\mathrm{ON}}$ and $u_{\mathrm{OFF}}$ gives the orbit segment that characterises the solution of interest. An illustration of this idea is given in Figure 2, where $u_{\mathrm{ON}}$ is the segment coloured red, and $u_{\mathrm{OFF}}$ is the segment coloured blue. Both $u_{\mathrm{ON}}$ and $u_{\mathrm{OFF}}$ are solution segments of (1) but for different values of $I_{\mathrm{app}}$ and for different integration times $T_{\mathrm{ON}}$ and $T_{\mathrm{OFF}}$, respectively. Using the set-up that is standard in Auto[55,56], we formulate a boundary value problem using scaled equations such that the total integration time for both segments is 1. That is, $u_{\mathrm{ON}}$ and $u_{\mathrm{OFF}}$ are solutions of

(5) $$u_{\mathrm{ON}}^{\prime }(t)=T_{\mathrm{ON}}\;f\left(u_{\mathrm{ON}}(t),\lambda ,I_{\mathrm{app}}\right),$$

(6) $$u_{\mathrm{OFF}}^{\prime }(t)=T_{\mathrm{OFF}}\;f\left(u_{\mathrm{OFF}}(t),\lambda ,0\right).$$

In order to obtain a unique solution pair $\left(u_{\mathrm{ON}},u_{\mathrm{OFF}}\right)$, we must impose boundary conditions. The boundary conditions for (5) are determined by the fact that the current injection perturbs system (1) from its resting potential for a fixed duration $T_{\mathrm{ON}}$, as indicated by horizontal black and vertical red dashed lines. Hence, (5) is effectively an initial value problem with $u_{\mathrm{ON}}(0)=u_{\mathrm{OFF}}^{*}$, where $u_{\mathrm{OFF}}^{*}$ is the stable equilibrium of (1) with $I_{\mathrm{app}}=0$; we solve for $u_{\mathrm{OFF}}^{*}$ implicitly in Auto[55,56], and the boundary condition becomes

(7) $$f\left(u_{\mathrm{ON}}(0),\lambda ,0\right)=0.$$

Equations (5) and (7) uniquely define the orbit segment $u_{\mathrm{ON}}$ as a function of *λ* for fixed $T_{\mathrm{ON}}$. The orbit segment $u_{\mathrm{OFF}}$ continues on from $u_{\mathrm{ON}}$, but now, $I_{\mathrm{app}}=0$. Hence, $u_{\mathrm{OFF}}$ is again effectively the solution of an initial value problem with initial condition

(8) $$u_{\mathrm{OFF}}(0)=u_{\mathrm{ON}}(1).$$

Throughout this paper, we use $I_{\mathrm{app}}=20\;\mu {\text{A/cm}}^{2}$ for a total duration $T_{\mathrm{ON}}=3\text{ ms}$, which is long enough to drive the system past its threshold for the constants as in Table 1. We fix $T_{\mathrm{OFF}}=297\text{ ms}$ so that the total integration time of the orbit segment is $T_{\mathrm{ON}}+T_{\mathrm{OFF}}=300\text{ ms}$, which is long enough for $u_{\mathrm{OFF}}(1)$ to be (approximately) at the resting potential. System (5)-(8) is now well posed and uniquely defines a *λ*-dependent solution family. 

**Fig. 3:**
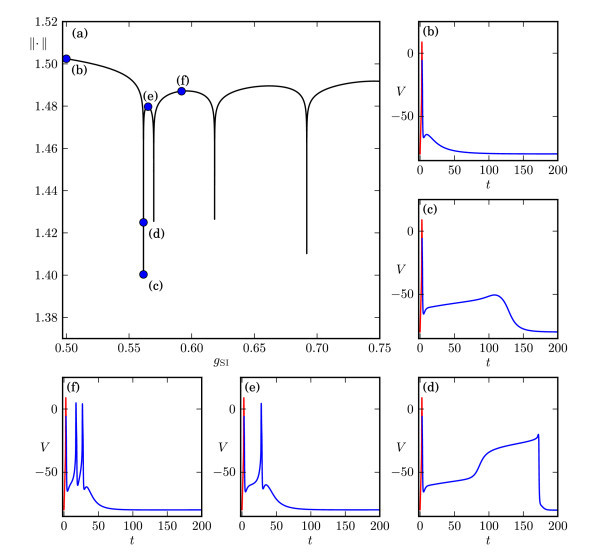
Continuation for increasing $g_{\mathrm{SI}}$ of solutions to system (5)-(8). Panel **(a)** shows the Auto$L_{2}$-norm <abbrgrp>^55^,^56^</abbrgrp> of the solution branch versus $g_{\mathrm{SI}}$ and illustrates that the spike-adding mechanism happens suddenly via a pronounced drop in norm; panels **(b)(c)(d)(e)** and **(f)** show representative solutions along the branch, indicated by the correspondingly *labelled dots* in panel (a), and illustrate that solutions during a spike generation, i.e., panels (c) and (d), exhibit a stretched ADP that develops into a double step before relaxing back to the resting potential.

 As illustrated by the example in Figure 1, we expect that increasing $g_{\mathrm{SI}}$ leads to a spike-adding transition; a new spike is added on top of ADP when it reaches a critical threshold of the membrane potential *V*. Hence, we set $\lambda =g_{\mathrm{SI}}$ in (5)-(8) and solve it by continuation in Auto[55,56], starting from $g_{\mathrm{SI}}=0.5{\text{ mS/cm}}^{2}$. Figure 3a shows the resulting solution branch using the standard $L_{2}$-norm of Auto[55,56] as a measure. We observe that the solution norm exhibits a series of fairly constant ‘plateaus’ that are separated by sharp downward peaks. This behaviour seems similar to that of spike-adding phenomena of periodic bursting solutions, which is organised by pairwise saddle-node bifurcations of periodics [19,26]. However, our numerical set-up imposes a fixed initial condition rather than a periodicity constraint. Hence, the uniqueness of the solutions of (5)-(8) prevents the possibility of coexisting orbit segments, that is, the branch in Figure 3a cannot have folds with respect to $g_{\mathrm{SI}}$. The orbit segments of selected solutions along the branch are shown in Figure 3b,c,d,e,f; note that we present the time series up to t=200 ms for clarity of the presentation. 

 Figure 3b shows our starting solution, i.e., a single spike followed by ADP. Along the first plateau of the solution branch up to the first downward peak, all orbit segments are qualitatively like Figure 3b; in particular, the ADP is a small hump. As we follow the solutions into the downward peak, the hump of the ADP for the orbit segments stretches out as shown in Figure 3c, which lies at the bottom of the downward peak. Interestingly, as we follow the solution back up along the downward peak, the orbit segment generates a double step in the ADP, as shown in Figure 3d; we selected the orbit segment with the longest double step (with respect to time). As we continue to trace the solution up along the downward peak, the small spike at the end of the orbit segment grows into a fully developed spike, while the stretched double step retracts; the orbit segment shown in Figure 3e is representative of such a solution, and all orbit segments along the second plateau in Figure 3a are qualitatively like Figure 3e. Figure 3f represents a solution along the next plateau, which exhibits three spikes that are created via the same process as explained above for the two-spike burst. In fact, the same spike-adding process takes place for all spike-adding transitions via the downward peaks in Figure 3a. We emphasise here that the stretched single- and double-step ADPs, as shown in Figure 3c,d, only exist along the downward peaks in Figure 3a, that is, in the exponentially small parameter interval during which a spike-adding transition occurs. Hence, such solutions are unlikely to be observed in actual experiments, and they are also very difficult to find in numerical experiments that use initial-value integration methods. The fact that a spike-adding transition happens over an exponentially small parameter interval, during which the solution measure changes rapidly, suggests that it is organised by the difference in time scales present in system (1). Therefore, in order to obtain a better understanding, we proceed by using GSPT [34-36,41,62,63].

## 4 Spike-adding organised by the critical manifold

As mentioned in Section 2, the full five-dimensional system (1) contains a three-dimensional fast subsystem with variables *V*, $m_{\mathrm{SI}}$ and $m_{\mathrm{FO}}$. Since $m_{\mathrm{SO}}$ and $h_{\mathrm{SI}}$ are much slower, the idea of GSPT is to assume that $m_{\mathrm{SO}}$ and $h_{\mathrm{SI}}$ do not change at all and treat them as parameters. More precisely, we consider the *singular limit* of system (1) and analyse the dynamics of the layer equation

$$\frac{du}{dt}=\frac{d}{dt}\left(\begin{array}{c} V\\ m_{\mathrm{SI}}\\ m_{\mathrm{FO}}\\ m_{\mathrm{SO}}\\ h_{\mathrm{SI}} \end{array}\right)=\left(\begin{array}{c} f_{1}(u,\lambda ,I_{\mathrm{app}})\\ f_{2}(u,\lambda )\\ f_{3}(u,\lambda )\\ 0\\ 0 \end{array}\right).$$

Furthermore, because we are interested in spike-adding phenomena after the brief current injection, we set $I_{\mathrm{app}}=0$.

 The important objects of study in the singular limit are equilibria and periodic orbits. Since $m_{\mathrm{SO}}$ and $h_{\mathrm{SI}}$ are parameters, these invariant objects occur in two-parameter families. The $\left(m_{\mathrm{SO}},h_{\mathrm{SI}}\right)$-dependent families of equilibria are known as the *critical manifold*, which we denote by *S*. The equilibria on *S* can be stable or unstable, determined with respect to the three-dimensional fast subsystem, and are typically separated by curves of fold or Hopf bifurcations. Similarly, we can expect the existence of $\left(m_{\mathrm{SO}},h_{\mathrm{SI}}\right)$-dependent families of periodic orbits that emanate from a curve of Hopf bifurcations on the critical manifold; these periodic orbits can again be stable or unstable with respect to the three-dimensional fast subsystem. Typically, the stable periodic orbits of the fast subsystem organise the spiking phase of the bursting oscillators [16,19,26].

 The critical manifold *S*, when considered in the full five-dimensional phase space of system (1), is a two-dimensional surface, or collection of surfaces, and the associated families of periodic orbits form a three-dimensional manifold, or collection of manifolds, that we denote by *P*. Together, these collections of manifolds organise the behaviour of solutions of (1). If $m_{\mathrm{SO}}$ and $h_{\mathrm{SI}}$ vary slowly enough, then GSPT guarantees that a solution of (1) (with $I_{\mathrm{app}}=0$) will trace attracting sheets of *S* or *P* that correspond to the $\left(m_{\mathrm{SO}},h_{\mathrm{SI}}\right)$-dependent families of attractors of the fast subsystem [41]. For example, the transient spikes of system (1) trace the manifold $P^{a}$ that corresponds to the family of attracting periodic orbits of the fast subsystem, while $m_{\mathrm{SO}}$ and $h_{\mathrm{SI}}$ are slowly varying [16,64]. More precisely, solutions of (1) lie on so-called slow manifolds that are perturbations of the different sheets of *S* and *P* from the singular limit [41]. Solutions of (1) are characterised by fast transitions between, followed by exponential contraction onto the slow manifolds. The essential difference in behaviour during a spike-adding transition is the fact that the solution of (1) contains a segment that traces a slow manifold associated with a sheet of *S* that is unstable (of saddle type) rather than attracting; see also [25,34,36,63]. While technically the solutions of (1) trace slow manifolds, we will abuse notation and write ‘sheet of *S*’ where we mean ‘slow manifold corresponding to the sheet of *S*.’

**Fig. 4:**
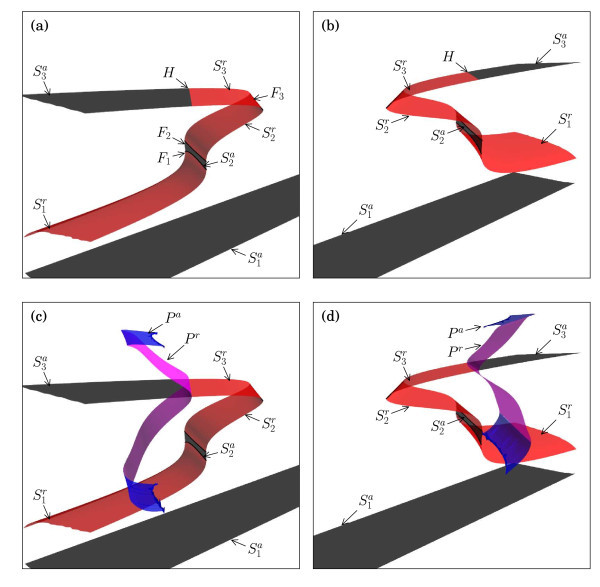
Critical manifolds for $g_{\mathrm{SI}}=0.5615{\text{ mS/cm}}^{2}$ embedded in the five-dimensional phase space of system (1). Shown are projections onto the $\left(h_{\mathrm{SI}},m_{\mathrm{SO}},V\right)$-coordinates; panels **(a)** and **(b)** show two different viewpoints of the surfaces of equilibria, *coloured black* when stable and *red* when not; from the same viewpoints, panels **(c)** and **(d)** also show maxima and minima with respect to *V* of the two-parameter families of periodic orbits, *coloured blue* when stable and *magenta* when not. The equilibrium manifold splits into six sheets, labelled $S_{1}^{a}$, $S_{1}^{r}$, $S_{2}^{a}$, $S_{2}^{r}$, $S_{3}^{r}$ and $S_{3}^{a}$, that are separated by four *fold curves*, $F_{0}$ (not shown), $F_{1}$, $F_{2}$ and $F_{3}$, and a curve of Hopf bifurcations labelled *H*. The saddle and attracting families of periodic orbits are labelled $P^{r}$ and $P^{a}$, respectively. See also Table 2.

 The geometry of *S* and *P* depends on the values of the other parameters in the system, such as the conductance $g_{\mathrm{SI}}$. In order to illustrate the spike generation, we consider the fast subsystem at the fixed value $g_{\mathrm{SI}}=0.5615{\text{ mS/cm}}^{2}$, which is approximately at the first downward peak in Figure 3a where the solution changes from a one-spike to a two-spike transient burst. Figure 4 shows the critical manifold *S* for this value of $g_{\mathrm{SI}}$ from two different viewpoints; in both views, the embedding into the five-dimensional phase space of (1) is projected onto the $\left(h_{\mathrm{SI}},m_{\mathrm{SO}},V\right)$-coordinates. The surface was obtained as follows: for ten fixed values of $m_{\mathrm{SO}}$ uniformly distributed in the interval [0,0.4], we computed the $h_{\mathrm{SI}}$-dependent curves of equilibria via standard equilibrium continuation with Auto[55,56], where we allowed $h_{\mathrm{SI}}$ to extend outside its physiological range of [0,1]; the surface *S* was obtained via concatenation of this collection of ten $m_{\mathrm{SO}}$-slices, and it is shown in Figure 4 with $h_{\mathrm{SI}}$ restricted to the interval [−1,1] for the sake of presentation. 

The critical manifold *S* in Figure 4 forms a single manifold, containing four fold curves, and can be divided into six different sheets depending on the stability type of the equilibria; the stable sheets are coloured black and the unstable ones red. The bottom (black) sheet is labelled $S_{1}^{a}$, and it contains the resting potential as a stable equilibrium on *S* that is an actual equilibrium of the full five-dimensional system (1) with $I_{\mathrm{app}}=0$. The sheet $S_{1}^{a}$ is connected via a curve $F_{0}$ of fold bifurcation points to the sheet labelled $S_{1}^{r}$ in Figure 4; this fold curve $F_{0}$ lies outside $h_{\mathrm{SI}}\in [-1,1]$ and is not shown in Figure 4. The sheet $S_{1}^{r}$ is a two-parameter family of equilibria with two stable and one unstable eigenvalues. Hence, $S_{1}^{r}$ has a four-dimensional stable and a three-dimensional unstable manifold. The next two sheets labelled $S_{2}^{a}$ and $S_{2}^{r}$ have the same stability types as $S_{1}^{a}$ and $S_{1}^{r}$, respectively; $S_{2}^{a}$ is connected to $S_{1}^{r}$ via the fold curve $F_{1}$ and $S_{2}^{a}$ and $S_{2}^{r}$ are separated by the fold $F_{2}$. Note that the sheet $S_{2}^{a}$ is nearly vertical (with respect to *V*), as shown in Figure 4; this is not an artefact of the chosen projection. The sheet $S_{3}^{r}$ is connected to $S_{2}^{r}$ via the fold curve $F_{3}$, and this sheet consists of equilibria with one stable and two unstable eigenvalues, that is, $S_{3}^{r}$ has a three-dimensional stable and a four-dimensional unstable manifold. The sheet $S_{3}^{r}$ ends at the curve *H* of Hopf bifurcations, after which it becomes stable again and is labelled $S_{3}^{a}$. An overview of the different sheets and their stability properties is provided in Table 2. 

**Table 2:** Stability properties of the critical manifold *S* and the manifold *P* of periodic orbits

	Two-dimensional critical manifold *S*						Three-dimensional manifold *P*	
	$S_{1}^{a}$	$S_{1}^{r}$	$S_{2}^{a}$	$S_{2}^{r}$	$S_{3}^{r}$	$S_{3}^{a}$	$P^{r}$	$P^{a}$
Dimension stable manifold	5	4	5	4	3	5	4	5
Dimension unstable manifold	N/A	3	N/A	3	4	N/A	4	N/A

Overview of the different sheets and stability properties of the critical manifold *S* and the manifold *P* of periodic orbits for the simplified model (1) with $g_{\mathrm{SI}}=0.5615{\text{ mS/cm}}^{2}$; the number of sheets, as well as their stability, does not change for any of the other choices of $g_{\mathrm{SI}}$ considered in this paper.

The maxima and minima of the families of periodic orbits originating from *H* are shown in Figure 4c,d, using the same two viewpoints as in panels (a) and (b), respectively. The Hopf bifurcation is subcritical along the entire curve so that the emanating family of periodic orbits is unstable (of saddle type), with four-dimensional stable and unstable manifolds; we coloured this family magenta and labelled it $P^{r}$. The family of periodic orbits becomes stable via a fold of periodic orbits, after which it is coloured blue and labelled $P^{a}$, and ends in a homoclinic bifurcation involving equilibria on the sheet $S_{1}^{r}$. We refer again to Table 2 for an overview of the different families and their stability properties.

**Fig. 5:**
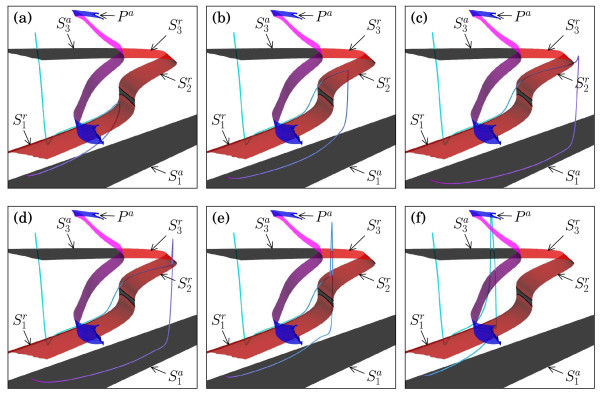
Orbit segments $u_{\mathrm{OFF}}(t)$ of the boundary value problem (5)-(8). Orbit segments $u_{\mathrm{OFF}}(t)$ for $g_{\mathrm{SI}}\approx 0.5615{\text{ mS/cm}}^{2}$ overlayed on the critical manifolds of Figure 4 with $g_{\mathrm{SI}}=0.5615{\text{ mS/cm}}^{2}$. The orbit segments are selected along the first downward peak in Figure 3a; panel **(a)** shows an orbit segment just before the minimum of the peak in Figure 3a is reached; panel **(b)** shows one shortly after; panel **(c)** shows the orbit segments labelled (d) in Figure 3a; and panels **(d)**, **(e)** and **(f)** show the spike generation as the orbit segments are continued until the start of the next ‘plateau’ in Figure 3a.

 Figure 5 illustrates how orbit segments selected from the first downward peak in Figure 3a trace the different sheets of the critical manifold *S* for $g_{\mathrm{SI}}=0.5615{\text{ mS/cm}}^{2}$; these orbit segments are all for virtually the same values $g_{\mathrm{SI}}\approx 0.5615{\text{ mS/cm}}^{2}$ that differ only in the seventh decimal point, during which the manifolds *S* and *P* hardly change at all. This extreme sensitivity of $g_{\mathrm{SI}}$ is a serious challenge for numerical computations, which we overcome by using continuation in Auto[55,56] of the orbit segments defined by (5)-(8). For clarity of the presentation, we show only the segment $u_{\mathrm{OFF}}(t)$, that is, after the current injection, depicted as a blue gradient (cyan to magenta) to visualise its evolution in time. In each panel of Figure 5, the orbit segment starts from $u_{\mathrm{OFF}}(0)=u_{\mathrm{ON}}(1)$, which is located at the top-left in each panel, above the sheet $S_{3}^{a}$; since $g_{\mathrm{SI}}$ hardly changes, $u_{\mathrm{OFF}}(0)$ is virtually the same point in all of these panels. The orbit segments $u_{\mathrm{OFF}}(t)$ traverse the critical manifold *S* before reaching the stable equilibrium of the full system (1), which lies on the bottom stable sheet $S_{1}^{a}$. Recall that the full phase space is five dimensional, and Figure 5 may show intersections that are due to projection onto the $\left(h_{\mathrm{SI}},m_{\mathrm{SO}},V\right)$-coordinates; Figure 5 gives the best possible projection and viewpoint to illustrate the location of the one-dimensional orbit segments relative to *S*. Some anomalous intersections remain at isolated points, e.g., the intersection with the sheet $S_{3}^{a}$ of *S*, but the observation that $u_{\mathrm{OFF}}(t)$ traces sheets of *S* is real, also in the full five-dimensional space. 

The spike-adding process occurs along the downward peak in Figure 3a, during which the parameter $g_{\mathrm{SI}}$ remains almost fixed, but the orbit segments of system (5)-(8) change dramatically. We observe the formation of a stretched ADP, which initially gets increasingly longer and shortens again as we follow the orbit segments along the downward peak in Figure 3a. This transition is initiated by the fact that, at the special value $g_{\mathrm{SI}}\approx 0.5615{\text{ mS/cm}}^{2}$, the injected current perturbs the orbit segment such that $u_{\mathrm{ON}}(1)=u_{\mathrm{OFF}}(0)$ lies almost on the four-dimensional stable manifold of the saddle-unstable sheet $S_{1}^{r}$; more precisely, $S_{1}^{r}$ has a corresponding slow manifold with a corresponding finite-time stable manifold that $u_{\mathrm{ON}}(1)=u_{\mathrm{OFF}}(0)$ comes close to. The closer $u_{\mathrm{ON}}(1)=u_{\mathrm{OFF}}(0)$ lies to this four-dimensional manifold, the closer the corresponding orbit segment comes to the slow manifold associated with $S_{1}^{r}$ and the longer it will trace this slow manifold. We approximate the slow manifold by the critical manifold $S_{1}^{r}$, and Figure 5a shows the orbit segment from Figure 3c, which traces $S_{1}^{r}$ almost up to the fold $F_{1}$ before it drops down to $S_{1}^{a}$ and converges to the resting potential.

 Figure 5b,c,d,e,f illustrates orbit segments for the second upward part of the downward peak in Figure 3a. Interestingly, a double-step ADP is created via a transition from $S_{1}^{r}$ to $S_{2}^{r}$ as shown. The orbit that was previously shown in Figure 3d traces both saddle-unstable sheets $S_{1}^{r}$ to $S_{2}^{r}$ of *S* all the way up to the fold $F_{3}$. This transition from $S_{1}^{r}$ to $S_{2}^{r}$ is, in fact, a robust part of the spike-adding process that is a continuous (parameter-dependent) variation of the case where the folds $F_{1}$ and $F_{2}$ are absent, that is, where $S_{1}^{r}$ connects directly to $S_{3}^{r}$; see Section 4.2 for more details. After reaching the top fold $F_{3}$, the membrane potential *V* initially increases instead of immediately decreasing down to the stable sheet $S_{1}^{a}$, and a small spike is created. As we continue to follow the solution up along the downward peak, the spike part of the orbit segment grows and moves back towards the attracting periodic orbit family $P^{a}$, as illustrated in Figure 5d,e. Finally, as shown in Figure 5f, the orbit segment traces $S_{1}^{r}$ for only a very short time before the second spike occurs; this orbit segment is selected almost at the end of the downward peak, after which orbit segments stop tracing $S_{1}^{r}$ altogether, and the transition from a one- to two-spike transient burst ends. We refer to Govaerts and Dhooge [24], Guckenheimer and Kuehn [25], and Osinga et al. [65] for similar transitions of periodic bursting oscillators.

 We remark here that the manner of eventual convergence to the resting potential depends on the nature of the lift-off from the slow manifolds that correspond to the sheet $S_{1}^{r}$ or $S_{2}^{r}$. Recall that both sheets $S_{1}^{r}$ and $S_{2}^{r}$ of the critical manifold *S* have three-dimensional stable manifolds; see Table 2. This means that the associated slow manifolds have a one-dimensional repelling fast component [41], and orbit segments that trace these saddle-unstable slow manifolds can leave it only along a single fast direction. We can see this in Figure 5 as a lift-off from $S_{1}^{r}$ ‘down’ in *V*, shown in Figure 5a, or a lift-off from $S_{1}^{r}$ ‘up’ in *V*, shown in Figure 5f; this uniquely defined change in direction along the one-dimensional repelling fast component is real and not just due to the projection onto $\left(h_{\mathrm{SI}},m_{\mathrm{SO}},V\right)$-space. The same holds for the sheet $S_{2}^{r}$, for which Figure 5b,e is a good example that also shows the required lift-off up from $S_{1}^{r}$ in order to reach $S_{2}^{r}$. In what follows, the notions up and down are with respect to this uniquely-defined change in direction.

The behaviour of the orbit segment of system (1) in relation to the critical manifold *S* of the fast subsystem that corresponds to the first downward peak in Figure 3a is representative for what happens along the other downward peaks in Figure 3a. Each time $g_{\mathrm{SI}}$ reaches a special value such that the orbit segment comes close enough to the four-dimensional stable manifold of $S_{1}^{r}$, it gets trapped onto $S_{1}^{r}$ (or, more precisely, the corresponding saddle-unstable slow manifold) for increasingly longer times, and the next spike-adding transition begins. For the parameters of Table 1, we found that this process always includes a transition between two saddle-unstable sheets, which organises the double-step ADP solutions. As mentioned before, the two sheets $S_{1}^{r}$ and $S_{2}^{r}$ together are perturbations of a single sheet connected to $F_{3}$ that can be obtained continuously via a small parameter variation (using a suitable parameter from Table 1), such that the fold curves $F_{1}$ and $F_{2}$ disappear in a curve of cusp bifurcations. Therefore, in the next section, we first explain the jump at the end of the canard-like behaviour, that is, the behaviour near the fold $F_{3}$ that separates the two saddle-unstable sheets $S_{2}^{r}$ and $S_{3}^{r}$. We then discuss the transition between $S_{1}^{r}$ and $S_{2}^{r}$ in Section 4.2. Section 4.3 illustrates how the spike-adding mechanism can change when additional equilibria are present.

### 4.1 Slow flow on the critical manifold near $F_{3}$

 Let us first focus our attention on the behaviour near the fold $F_{3}$, that is, the transition from Figure 5c to Figure 5d. The behaviour near folds can be explained by analysis of the slow flow on the critical manifold *S*[63]. The slow flow on *S* is defined by the differential algebraic system

(9) $$\left(\begin{array}{c} 0\\ 0\\ 0\\ {\dot{m}}_{\mathrm{SO}}\\ {\dot{h}}_{\mathrm{SI}} \end{array}\right)=\left(\begin{array}{c} f_{1}(u,\lambda )\\ f_{2}(u,\lambda )\\ f_{3}(u,\lambda )\\ f_{4}(u,\lambda )\\ f_{5}(u,\lambda ) \end{array}\right).$$

Here, we always have $I_{\mathrm{app}}=0$. Recall that the gating variables of (1) are only coupled through the membrane potential *V*. In fact, it is easy to solve equations $f_{2}(u,\lambda )=0$ and $f_{3}(u,\lambda )=0$ explicitly, which gives us the solutions for the fast gating variables $m_{\mathrm{SI}}=m_{{\mathrm{SI}}_{\infty }}(V)$ and $m_{\mathrm{FO}}=m_{{\mathrm{FO}}_{\infty }}(V)$. We substitute these solutions into $f_{1}$ to obtain

$$\left(\begin{array}{c} 0\\ {\dot{m}}_{\mathrm{SO}}\\ {\dot{h}}_{\mathrm{SI}} \end{array}\right)=\left(\begin{array}{c} f_{1}^{*}(V,m_{\mathrm{SO}},h_{\mathrm{SI}},\lambda )\\ f_{4}^{*}(V,m_{\mathrm{SO}},\lambda )\\ f_{5}^{*}(V,h_{\mathrm{SI}},\lambda ) \end{array}\right),$$

that is, the slow flow on the two-dimensional critical manifold *S* is defined by two ordinary differential equations for $m_{\mathrm{SO}}$ and $h_{\mathrm{SI}}$ and a single algebraic constraint $f_{1}^{*}(V,m_{\mathrm{SO}},h_{\mathrm{SI}},\lambda )=0$. Unfortunately, *S* is folded with respect to *V* so that $m_{\mathrm{SO}}$ and $h_{\mathrm{SI}}$ do not uniquely define *V*; however, the algebraic constraint does uniquely define $m_{\mathrm{SO}}$ or $h_{\mathrm{SI}}$ from given pair $\left(V,m_{\mathrm{SO}}\right)$ or $\left(V,h_{\mathrm{SI}}\right)$, respectively; compare also Figure 4a,b. Hence, it is advantageous to express the slow flow in terms of only one of the slow variables, $m_{\mathrm{SO}}$ or $h_{\mathrm{SI}}$, together with the fast variable *V*.

 We choose to work with *V* and $m_{\mathrm{SO}}$. If we formally differentiate the algebraic constraint, we obtain (10)

 where $h_{\mathrm{SI}}$ is uniquely determined from $f_{1}^{*}(V,m_{\mathrm{SO}},h_{\mathrm{SI}},\lambda )=0$. We refer to Desroches et al. [63] for more details on this step. Note that (10) becomes singular when $\partial f_{1}^{*}/\partial V=0$, that is, precisely where *S* has folds with respect to *V*. We can desingularise the flow by scaling time with the factor $-\partial f_{1}^{*}/\partial V$. This rescaling reverses the direction of the time whenever $\partial f_{1}^{*}/\partial V>0$, and we obtain the desingularised slow flow in the form (11)

**Fig. 6:**
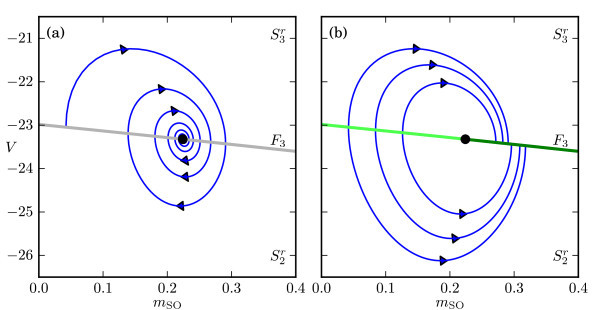
Phase portraits on the sheets $S_{2}^{r}$ and $S_{3}^{r}$ of the critical manifold *S* near the fold $F_{3}$. Panel **(a)** shows a projection onto the $\left(m_{\mathrm{SO}},V\right)$-plane of a trajectory of the desingularised slow flow (11), which converges to an attracting focus on $F_{3}$ (*grey line*), and panel **(b)** shows projected trajectories of the slow flow (9). The repelling and attracting nature of $F_{3}$ is indicated by *light*- and *dark-greencolours*, respectively.

**Fig. 7:**
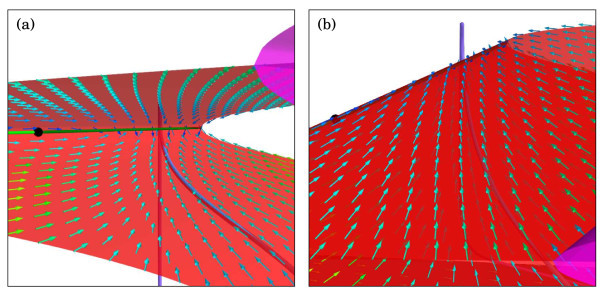
The slow flow on *S* near the top fold $F_{3}$. Vectors of the slow flow on *S* are shown together with the orbit segment from Figure 5c. The direction of flow on *S* is indicated by the arrows where *hotter colours* correspond to vectors with larger magnitudes. The folded focus is the *black dot* on $F_{3}$ with the uniformly repelling and attracting parts of $F_{3}$*coloured light* and *dark green*, respectively. Panels **(a)** and **(b)** show the process from two opposite viewpoints, and panels **(c)** and **(d)** show corresponding close-up views near $F_{3}$.

 The actual slow flow on *S* is now defined by the desingularised slow flow (11) where we must take into account the time reversal in the regimes where $\partial f_{1}^{*}/\partial V>0$. Figure 6 illustrates this for a neighbourhood of the fold $F_{3}$ on *S* that separates the sheets $S_{2}^{r}$ and $S_{3}^{r}$; we have $\partial f_{1}^{*}/\partial V<0$ on $S_{3}^{r}$ and $\partial f_{1}^{*}/\partial V>0$ on $S_{2}^{r}$. The phase portraits in Figure 6 are projected onto the $\left(m_{\mathrm{SO}},V\right)$-plane. Figure 6a shows how a trajectory of (11) near $F_{3}$ (grey line) is attracted to a focus equilibrium of the desingularised slow flow, marked with a black dot on $F_{3}$. Figure 6b shows the corresponding projection of the slow flow (9) on *S*; note the change in direction of the flow for the region where $\partial f_{1}^{*}/\partial V>0$. The fold $F_{3}$ in Figure 6b is now divided into two parts, a repelling segment on the left side of the focus equilibrium (light-green line) and an attracting segment on the right side of the focus equilibrium (dark-green line). In fact, the focus equilibrium is no longer a focus; it has become a folded singularity or, more precisely, a folded focus. We refer to Wechselberger [36] and Desroches et al. [63] for more details. Figure 7 shows the sheets $S_{2}^{r}$ and $S_{3}^{r}$ of the critical manifold near the folded singularity, with the orbit segment from Figure 5c depicted by a blue-gradient curve as before; panels (a) and (b) provide two opposite viewpoints, and panels (c) and (d), two corresponding close-up views. The slow flow is visualised as a vector field on *S*, where hotter colours depict vectors with a higher magnitude (the length of the vectors is constant for clarity of presentation). The fold $F_{3}$ in Figure 7 is coloured the same dark and light green as in Figure 6b. 

Figure 7a,b shows a projection onto $\left(h_{\mathrm{SI}},m_{\mathrm{SO}},V\right)$-coordinates of how the orbit segment follows the slow flow on $S_{2}^{r}$ as it approaches $F_{3}$. The first part of the orbit segment lies slightly above (relative to this projection) $S_{1}^{r}$, and after a jump, another part of this orbit segment lies slightly below $S_{2}^{r}$. In a neighbourhood of the folded focus on the fold curve $F_{3}$, the slow flow has the form of large semi-cycles that cause the orbit segment to trace $S_{2}^{r}$ laterally and, at the same time, push it toward $F_{3}$. Since the flow on the top sheet $S_{3}^{r}$ also points towards $F_{3}$, as shown in Figure 7c,d, the orbit segment cannot pass $F_{3}$ and reaches a so-called jump point; compare also with Figure 6b. At the jump point, the fast directions of the flow take over, which causes the formation of a small spike as the orbit segment leaves *S*; see also Figure 5c. Let us emphasise here that the behaviour of the orbit segments near $F_{3}$ does not involve interactions with the slow flow on $S_{3}^{r}$; the small spike and subsequent drop down to $S_{1}^{a}$ do not intersect the surfaces $S_{3}^{r}$ and $S_{2}^{r}$. As mentioned earlier in this section, the actual spike formation develops as soon as an orbit segment has reached $F_{3}$. After reaching $F_{3}$, the orbit segment will lie slightly *above* (relative to this projection) $S_{2}^{r}$ and experience a lift-off up from $S_{2}^{r}$ (or later only from $S_{1}^{r}$ as shown in Figure 5f). The spike-formation takes place on the fast time scale, and any perceived intersections with $S_{3}^{r}$ and $S_{2}^{r}$ are due to the projection onto $\left(h_{\mathrm{SI}},m_{\mathrm{SO}},V\right)$-coordinates.

### 4.2 Slow flow of the critical manifold near the folds $F_{1}$ and $F_{2}$

 The formation of a new well-developed spike occurs over an exponentially small parameter interval $g_{\mathrm{SI}}\approx 0.5615{\text{ mS/cm}}^{2}$ for which the effect of the injected current is precisely such that the orbit segment comes close to the four-dimensional stable manifold of $S_{1}^{r}$. The behaviour of the orbit segment near the top fold $F_{3}$ corresponds to the onset of such a new spike, but the process of reaching $F_{3}$, as illustrated in Figure 5a,b, as well as the further development of the spike, as illustrated in Figure 5d,e,f, involves the creation of a double-step ADP; this behaviour is organised by a (fast) jump from $S_{1}^{r}$ to another saddle-unstable sheet $S_{2}^{r}$. Such a jump, which is actually a jump between the two corresponding saddle-unstable slow manifolds, is a phenomenon that occurs robustly as part of the spike-adding mechanism and has previously been observed for periodic orbits in planar systems; it was reported as a new type of canard called *fold-initiated canards* in a study by Guckenheimer et al. [45], and a slightly different version termed *ducks with relaxation* is discussed by Arnol’d [66], Ch.4, Sec.5.4]. In fact, the behaviour we observe in our model is essentially planar and very similar to the example discussed by Guckenheimer et al. [45]. Indeed, there are no folded singularities on the folds $F_{1}$ and $F_{2}$, which means that each fold point has the same effect on the dynamics, and the slow flow is essentially one dimensional. Furthermore, the repelling fast component of the slow manifolds associated with $S_{1}^{r}$ and $S_{2}^{r}$ is one dimensional as well.

**Fig. 8:**
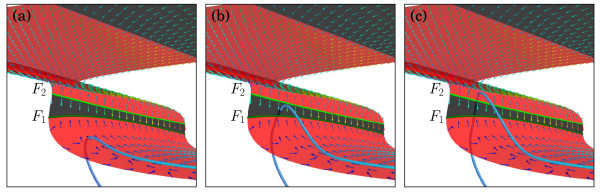
The slow flow on *S* in the vicinity of the two folds $F_{1}$ and $F_{2}$. The folds $F_{1}$ and $F_{2}$ mark the transition of the orbit segment between the two saddle-unstable sheets $S_{1}^{r}$ and $S_{2}^{r}$; the direction of flow on *S* is indicated by the arrows where *hotter colours* correspond to vectors with larger magnitudes. The attracting fold $F_{1}$ is *dark green*, and the repelling fold $F_{2}$ is *light green*. Panels **(a)**, **(b)** and **(c)** show the same perspective with orbit segments that almost reach $F_{1}$, reach $F_{1}$ via the attracting sheet $S_{2}^{a}$ and reach $F_{2}$ with a jump from $S_{1}^{r}$ to $S_{2}^{r}$, respectively.

The presence of folds $F_{1}$ and $F_{2}$ results in the formation of a double-step ADP during the spike-adding process; see Figure 5a,b. The double-step creation is a direct consequence of the fact that the direction of the slow flow on *S* is transverse to both fold curves $F_{1}$ and $F_{2}$; this is illustrated in Figure 8. Figure 8a shows the same orbit segment as in Figure 5a, and Figure 8b,c shows two subsequent orbit segments that both occur before the case shown in Figure 5b. As before, the orbit segments are depicted as blue-gradient curves, and the colour-coded vectors indicate the slow flow on *S*. We observe that the orbit segment in Figure 8a exhibits a lift-off down from $S_{1}^{r}$ before a fast jump down to $S_{1}^{a}$ returns the system to its resting potential, while the two orbit segments in Figure 8b,c exhibit a lift-off up from $S_{1}^{r}$. These orbit segments are all part of the same continuous one-parameter family of orbit segments that trace $S_{1}^{r}$; each orbit segment corresponds to a unique value of $g_{\mathrm{SI}}$ even though we always have $g_{\mathrm{SI}}\approx 0.5615{\text{ mS/cm}}^{2}$ and the variation is exponentially small, occurring only in the seventh decimal place. 

Let us consider this continuous one-parameter family of orbit segments as identified by the moment of lift-off (first down and then up) from $S_{1}^{r}$. At the start of the spike-adding process, orbit segments trace only the saddle-unstable sheet $S_{1}^{r}$ before a lift-off down to $S_{1}^{a}$ returns the system to its resting potential; the example in Figure 8a shows an orbit segment that almost reaches $F_{1}$. As $g_{\mathrm{SI}}\approx 0.5615{\text{ mS/cm}}^{2}$ increases continuously (but only exponentially small), the orbit segments come increasingly closer to $S_{1}^{r}$ until one actually reaches $F_{1}$; these orbit segments grow increasingly longer stretched ADPs.

Using the analysis via the desingularised slow flow (11) as derived in Section 4.1, we can decide what happens when an orbit segment reaches $F_{1}$. We find that the desingularised slow flow (11) does not have any equilibria in the neighbourhood of the two folds $F_{1}$ and $F_{2}$, which means that there are no folded singularities on either $F_{1}$ or $F_{2}$; the fold curve $F_{1}$ is uniformly attracting, which we indicated by a dark-green colour, and $F_{2}$ is uniformly repelling, indicated by a light-green colour. Hence, upon reaching $F_{1}$, the orbit segment simply jumps down toward the resting potential, and subsequent orbit segments exhibit a lift-off up from $S_{1}^{r}$. Since the sheet $S_{2}^{a}$ on the other side of $F_{1}$ is attracting, the fast directions will push these orbit segments toward $S_{2}^{a}$, provided that the lift-off up from $S_{1}^{r}$ occurs not too far away from $F_{1}$. Note that the slow flow on $S_{2}^{a}$ points back to $F_{1}$, so orbit segments that reach $S_{2}^{a}$ will flow to $F_{1}$ and again drop down to $S_{1}^{a}$; an example is shown in Figure 8b.

 As we continue to increase $g_{\mathrm{SI}}\approx 0.5615{\text{ mS/cm}}^{2}$ ever so slightly, orbit segments will converge to $S_{2}^{a}$ closer and closer near $F_{2}$, until the lift-off up from $S_{1}^{r}$ happens earlier, far enough from $F_{1}$, such that they may reach $F_{2}$. In order to determine what happens when an orbit segment reaches $F_{2}$, we must remember that system (1) and, hence, the slow flow on *S* depend continuously on $g_{\mathrm{SI}}$. This means that the family of orbit segments is also continuous, and the orbit segment that reaches $F_{2}$ is a continuous variation of an orbit segment that experiences a lift-off up from $S_{1}^{r}$ later so that it still converges to $S_{2}^{a}$ and flows back to $F_{1}$ as well as an orbit segment that experiences a lift-off up from $S_{1}^{r}$ earlier so that it misses $F_{2}$ altogether and forms a well-developed spike as illustrated in Figure 5f. Therefore, the family of orbit segments must include a subfamily of orbit segments that experience a lift-off from $S_{2}^{r}$. Just as for $S_{1}^{r}$, the lift-off will first be down as a continuous variation from the orbit segments that flow along $S_{2}^{a}$ and then up to transform into an orbit segment that misses $F_{2}$ altogether. This subfamily exists in a (doubly) exponentially small parameter regime of fold-initiated canard behaviour [45] and consists of orbit segments that exhibit a double-step ADP.

Another way to understand this phenomenon is in terms of singularity theory, which suggests that $S_{1}^{r}$ and $S_{2}^{r}$ are part of the same surface that unfolds a cusp singularity. Recall that the critical manifold *S* is a collection of families of equilibria of the fast subsystem of (1); the folds $F_{1}$ and $F_{2}$ are curves of saddle-node bifurcations that exist robustly in the $\left(m_{\mathrm{SO}},h_{\mathrm{SI}}\right)$-parameter plane organised by the two slow variables. Since $F_{1}$ and $F_{2}$ are typically not parallel, they will meet; note that $F_{1}$ and $F_{2}$ may need to be extended into an unphysical parameter regime of the $\left(m_{\mathrm{SO}},h_{\mathrm{SI}}\right)$-plane. Singularity theory tells us that the two fold curves $F_{1}$ and $F_{2}$ typically meet at a cusp point and end there, which means that the two sheets $S_{1}^{r}$ and $S_{2}^{r}$ merge into one in this region of the $\left(m_{\mathrm{SO}},h_{\mathrm{SI}}\right)$-plane. The existence of a double-step ADP then merely depends on the location relative to the cusp point of the interaction between the orbit segments and the critical manifold *S*. We remark that a small change in one or more of the parameters given in Table 1 may move the cusp point into the physical regime or such that $F_{1}$ and $F_{2}$ no longer exist for physiologically realistic values of $m_{\mathrm{SO}}$ and $h_{\mathrm{SI}}$; in the latter case, the spike-adding transition will not feature a double-step ADP.

### 4.3 Spike-adding when additional equilibria are present

It turns out that the spike-adding mechanism organised by canard-like behaviour during the downward peaks of Figure 3a always features a double-step ADP stage involving a jump between $S_{1}^{r}$ and $S_{2}^{r}$. Hence, each downward peak in Figure 3a corresponds to a qualitatively similar transition as discussed for the first one at $g_{\mathrm{SI}}\approx 0.5615{\text{ mS/cm}}^{2}$. If we increase $g_{\mathrm{FO}}$ from the fixed value $g_{\mathrm{FO}}=9.5{\text{ mS/cm}}^{2}$ that was used in Figure 3 to the new value $g_{\mathrm{FO}}=9.6{\text{ mS/cm}}^{2}$, then the nature of spike adding changes due to the presence of additional unstable equilibria of system (1). If we again continue the two-point boundary value problem (5)-(8) as before with $\lambda =g_{\mathrm{SI}}$ but $g_{\mathrm{FO}}=9.6{\text{ mS/cm}}^{2}$ set to its new value, we get a bifurcation diagram similar to the one for $g_{\mathrm{FO}}=9.5{\text{ mS/cm}}^{2}$ shown in Figure 3. In fact, the spike-adding mechanism for the first four additional spikes involves a double-step ADP stage as we have seen in the previous section. However, for $g_{\mathrm{SI}}\approx 0.7672{\text{ mS/cm}}^{2}$, that is, just before the transition from five to six spikes, a saddle-node bifurcation occurs on the saddle-unstable sheet $S_{1}^{r}$. This creation of two new (unstable) equilibria prevents a double-step ADP stage; the spike-adding mechanism only involves orbit segments exhibiting a stretched ADP with a singe step, and there is no longer a jump between saddle-unstable slow manifolds.

**Fig. 9:**
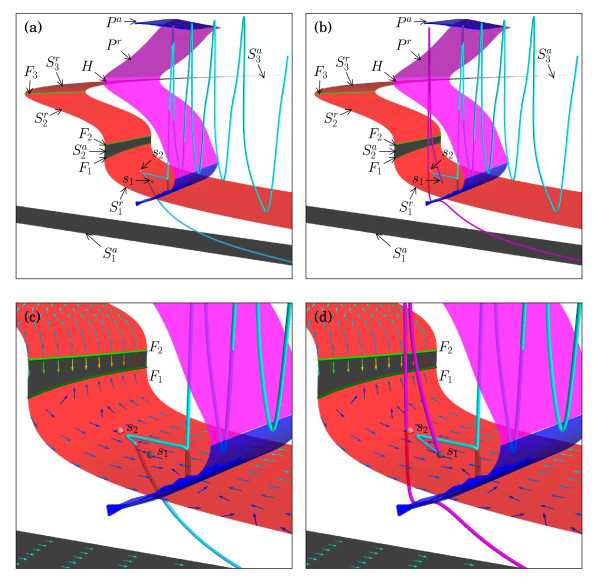
The critical manifold calculated for $g_{\mathrm{FO}}=9.6{\text{ mS/cm}}^{2}$ and $g_{\mathrm{SI}}=0.7842{\text{ mS/cm}}^{2}$ projected onto ($h_{\mathrm{SI}},m_{\mathrm{SO}},V$)-space. Superimposed in the left and right column are orbit segments with $g_{\mathrm{SI}}\approx 0.7842$ selected from the falling and rising slopes of the downward peak, respectively. Panels **(a)** and **(b)** show an overall view, and panels **(c)** and **(d)** are enlargements near $F_{1}$ and $F_{2}$ along with the associated slow flow. The two unstable equilibria $s_{1}$ and $s_{2}$ of the full system are marked with *black* and *red dots* because they are an attractor and a saddle on $S_{1}^{r}$, respectively.

Let us focus on the transition from a burst with five to one with six spikes, which takes place at $g_{\mathrm{SI}}\approx 0.7842{\text{ mS/cm}}^{2}$. For this value of $g_{\mathrm{SI}}$, there exists three equilibria, but only one is stable so that there is no bistability. The stable equilibrium is the resting potential on $S_{1}^{a}$. The other two equilibria are saddles, one with one and one with two unstable eigenvalues denoted $s_{1}$ and $s_{2}$, respectively; these additional saddle equilibria are located on $S_{1}^{r}$. We calculate the critical manifold *S* of the fast subsystem for $g_{\mathrm{SI}}=0.7842{\text{ mS/cm}}^{2}$; it is shown in Figure 9 projected onto ($h_{\mathrm{SI}},m_{\mathrm{SO}},V$)-space. Figure 9 illustrates that the critical manifold does not change qualitatively for higher values of $g_{\mathrm{FO}}$ and $g_{\mathrm{SI}}$; compare with Figure 4. Two orbit segments, one selected from the falling slope and one from the rising slope of the downward peak at $g_{\mathrm{SI}}\approx 0.7842{\text{ mS/cm}}^{2}$, are superimposed onto *S*; see Figure 9a,b with enlargements in Figure 9c,d, respectively. As before, only the part of the orbit segments that starts after the current injection is shown, so only the downward part of the first of the five spikes is visible. The enlargements in Figure 9c,d also show the slow flow on *S* in a neighbourhood of the two equilibria and better visualise the interaction of the two orbit segments with $s_{1}$ and $s_{2}$. The equilibria $s_{1}$ and $s_{2}$ are both saddles, but with respect to the slow flow on *S*, the equilibrium $s_{1}$ is stable (black dot) and $s_{2}$ is a saddle (red dot). 

The value $g_{\mathrm{SI}}\approx 0.7842{\text{ mS/cm}}^{2}$ for this case with $g_{\mathrm{FO}}=9.6{\text{ mS/cm}}^{2}$ is again special because at the end of the oscillations, when the orbit segment reaches the family of homoclinic orbits where $P^{a}$ ends, it lies extremely close to the four-dimensional stable manifold of $S_{1}^{r}$ so that it drops down and traces the saddle-unstable sheet $S_{1}^{r}$ of *S*. The difference with the spike-adding mechanism illustrated in Figure 5 is that the behaviour of the orbit segment on $S_{1}^{r}$ is affected by the presence of the equilibria $s_{1}$ and $s_{2}$. With respect to the two-dimensional slow flow on $S_{1}^{r}$, the equilibrium $s_{1}$ is an attractor, and all orbit segments on $S_{1}^{r}$ converge to $s_{1}$ provided that they lie in its basin of attraction, which is bounded by the one-dimensional stable manifold of the saddle $s_{2}$. In terms of the full five-dimensional flow, $S_{1}^{r}$ is obviously unstable, and orbit segments that come close enough to $S_{1}^{r}$ will behave as dictated by the slow flow for only a finite amount of time; this means that convergence to $s_{1}$ will eventually be followed by a fast repulsion away from $S_{1}^{r}$. The orbit segment in Figure 9a,c enters a close enough neighbourhood of $S_{1}^{r}$ in the region of the basin of attraction of $s_{1}$; hence, during the time that it is following the slow flow, it converges to $s_{1}$, but we can clearly see in Figure 9c that the fast directions take over before it reaches $s_{1}$. Since this orbit segment was selected from the falling slope of the downward peak of the spike-adding mechanism, the orbit segment jumps straight down toward $S_{1}^{a}$, where it converges to the resting potential. Orbit segments on this slope that lie closer to the minimum of the downward peak would come closer to $s_{1}$ but still jump down toward $S_{1}^{a}$ when the fast directions take over. On the other hand, orbit segments from the rising slope of the downward peak eventually experience a lift-off ‘up’ from $S_{1}^{r}$ so that a large action potential occurs before converging back to the resting potential; this change of direction corresponds to the onset of a new spike, which is more dramatic and abrupt than the gradual increase in *V* followed by a small-amplitude spike as illustrated in Figure 5.

Continuity of the vector field (1) implies that there exists an orbit segment that actually converges to the saddle $s_{1}$ and never relaxes back to the resting potential. This happens when $g_{\mathrm{SI}}\approx 0.7842{\text{ mS/cm}}^{2}$ is exactly at the value where the perturbed trajectory lies on the four-dimensional stable manifold $W^{s}(s_{1})$ of the equilibrium $s_{1}$. In contrast to the four-dimensional stable manifold of $S_{1}^{r}$, the manifold $W^{s}(s_{1})$ is invariant under the flow of (1), and this heteroclinic connection is a well-defined bifurcation for system (1).

We remark here that the presence of additional equilibria, such as $s_{1}$ and $s_{2}$ in the example discussed, only affects the spike-adding mechanism if the orbit segments that trace $S_{1}^{r}$ enter the basin of attraction of $s_{1}$. If such orbit segments trace $S_{1}^{r}$ on the other side of the stable manifold of $s_{2}$, then a double-step ADP stage would occur. We know from our further model analysis (not shown) that the unstable equilibria persists for higher values of $g_{\mathrm{SI}}$ as well as $g_{\mathrm{FO}}$, and in all cases that we investigated, these additional equilibria on $S_{1}^{r}$ affect the spike generation in the way described above.

## 5 Discussion

 In this paper, we performed a detailed analysis of the mechanisms of spike generation and spike-adding in a transient burst. Based on a reduction of our previous model [13], we identify these mechanisms using numerical continuation of orbit segments that are solutions to a well-posed boundary value problem. In our analysis, we utilised the separation of time scales in system (1). We calculated the two-dimensional critical manifold *S* of the fast subsystem, which organises the behaviour of the system. The spike-generation process is characterised by the fact that orbit segments trace saddle-unstable slow manifolds that correspond to saddle-unstable sheets of *S*. More precisely, there are two saddle-unstable sheets, $S_{1}^{r}$ and $S_{2}^{r}$, with four-dimensional stable and three-dimensional unstable manifolds; this means that the lift-off from the associated slow manifolds is characterised by a uniquely defined direction. The changes in sign of this direction mark the different phases of the spike-adding transition.

 By considering the slow flow on *S*, we were able to explain the onset of a spike as well as the double-step stretched ADP that leads up to it. For the value of $g_{\mathrm{FO}}=9.5{\text{ mS/cm}}^{2}$ that we considered, the onset of a spike is organised by the top fold $F_{3}$ of *S*. This fold contains a folded-focus singularity, but it is not accessible and the spikes are due to (regular) jump points. The folds $F_{1}$ and $F_{2}$ that are involved in the double-step ADP do not contain any folded singularities, and they are uniformly attracting and repelling, respectively. Therefore, the first step in the stretched ADP ends at a regular jump point. The second step occurs due to a type of fold-initiated canard because the slow flow points away from $F_{2}$; the fold-initiated canard-like behaviour forms a robust part of the spike-adding mechanism that has also been observed for periodic orbits [45]. The actual spike-adding transition occurs in an exponentially small parameter regime that is very difficult, if not impossible, to find with brute-force integration routines; therefore, it is also highly unlikely to observe anything like a stretched ADP or double-step ADP in experiments.

We found that the nature of the spike-adding mechanism may change if $g_{\mathrm{FO}}$ increases slightly. For higher values of $g_{\mathrm{FO}}$, an increase in $g_{\mathrm{SI}}$ causes the appearance of two equilibria, $s_{1}$ and $s_{2}$, on $S_{1}^{r}$ that form a saddle-node pair with respect to the slow flow. As it turns out, the presence of these equilibria prevents the double-step ADP. Instead, orbit segments that come close to $S_{1}^{r}$ during the spike-generation process flow towards the attracting equilibrium $s_{1}$ before a lift-off in the fast direction. This means that the onset of a spike is now organised by $s_{1}$ rather than the fold $F_{3}$, and the stretched ADP involves only a single step. A spike generated by $s_{1}$ is dramatically different from one generated by $F_{3}$. While both spike generations happen in an exponentially small parameter interval, the increase in amplitude of a spike generated by $F_{3}$ is gradual and should be viewed as a variation of the orbit segment that depends continuously on $g_{\mathrm{SI}}$. On the other hand, a spike generated by $s_{1}$ is not a continuous variation, and a large-amplitude spike appears abruptly as $g_{\mathrm{SI}}$ is increased (on an exponentially small scale). The two families, one with and one without the additional spike, are separated by a heteroclinic connection (via a current injection) from the resting potential to the saddle $s_{1}$; our numerical method for continuation of the family only gets past this discontinuity because we do not impose relaxation back to the resting potential but keep $T_{\mathrm{OFF}}$ fixed instead. Similar spike-adding mechanisms can be organised by the presence of a saddle periodic orbit or other saddle-type invariant object, but we did not observe this in our model.

In theory, it should be possible to have a double-step ADP as part of the spike-adding transition even when additional equilibria are present. The occurrence of a double-step ADP in this case only depends on whether tracing of $S_{1}^{r}$ commences in the basin of attraction of $s_{1}$ (restricted to $S_{1}^{r}$) or not, which is determined by the stable manifold of the saddle equilibrium $s_{2}$. In our numerical explorations, the orbit segments always commence tracing $S_{1}^{r}$ in the basin of attraction of $s_{1}$. Hence, we may conclude that $g_{\mathrm{FO}}$ and $g_{\mathrm{SI}}$ do not have a profound influence on the relative location where orbit segments begin to trace $S_{1}^{r}$ during the spike-adding process. However, other parameters of the system may alter this relative location and provoke a double-step ADP even in the presence of additional equilibria. While this observation indicates a challenge for a precise definition of spike-onset in our context, the two different mathematical notions seem to have the same biological effect.

We believe that the canard-like transition involving saddle-unstable sheets of the critical manifold lies at the heart of any spike-adding mechanism when no invariant saddle-type objects are present. The different phases during the transition, however, could be organised by features other than regular jump points and fold-initiated canards. For example, it should be expected that other folded singularities may appear due to variations in the slow flow on *S*. An investigation of all possibilities remains an interesting and challenging project for future work.

## Competing interests

The authors declare that they have no competing interests.

## Authors’ contributions

JN, HO, and KT performed the analysis and wrote or rewrote drafts of the manuscript. JN did all numerical computations and visualisations with assistance from HO and KT. All authors read and approved the final manuscript.
